# Up to One-Half of Runners Return to Running One Year After Arthroscopic Partial Meniscectomy

**DOI:** 10.1016/j.asmr.2022.06.002

**Published:** 2022-07-13

**Authors:** Eli T. Sayegh, Aseel G. Dib, Natalie A. Lowenstein, Jamie E. Collins, Rebecca G. Breslow, Elizabeth Matzkin

**Affiliations:** a Department of Orthopaedic Surgery, Brigham and Women’s Hospital, Boston, Massachusetts, U.S.A.; b University of Alabama Birmingham School of Medicine, Birmingham, Alabama, U.S.A.; c Department of Orthopaedic Surgery, Brigham and Women’s Hospital, Boston, Massachusetts, U.S.A.; d Harvard Medical School, Boston, Massachusetts, U.S.A.

## Abstract

**Purpose:**

To determine whether, and at which frequency, runners return to running after undergoing arthroscopic partial meniscectomy (APM).

**Methods:**

We identified patients who underwent surgery between August 2012 and December 2019 who were classified as runners (defined as running 2+ times per week according to Marx Activity Rating Scale Q1) and completed the 1-year follow-up to assess outcomes. Patients were followed using the Marx Activity Rating Scale, Knee Injury and Osteoarthritis Outcome Score (KOOS), Veterans RAND 12-item Health Survey mental and physical components, and visual analog pain scale scores preoperatively and 1 and 2 years postoperatively. The association between baseline characteristics and return to running was assessed using the unpaired *t* test or Wilcoxon rank sum test for continuous predictors and a χ^2^ test for categorical predictors, using the 1-year postoperative follow-up data.

**Results:**

A total of 185 patients were included in this study. One year after APM, 41% of runners returned to running at the same frequency or more frequently than before. Further, 50% of runners returned to running at least twice weekly. Return to running according to those definitions was similar at 2 years (38% and 47%, respectively). At both 1 and 2 years, runners exhibited significant improvements in KOOS (Pain), KOOS (Function in Sport and Recreation), visual analog pain scale, and Veterans RAND 12-item Health Survey physical component scores. Lower body mass index (*P* = .0248) and greater baseline running frequency (*P* = .0300) predicted return to running at least twice weekly at 1 year postoperatively. Medial versus lateral compartment partial meniscectomy and Outerbridge grade were not significant predictors of return to running.

**Conclusions:**

Roughly 1 in 2 runners return to their preoperative running frequency after undergoing APM. Obesity and lower baseline running frequency were significantly associated with inability to return to running.

**Level of Evidence:**

III, retrospective cohort study.

Running is among the most popular athletic pastimes in the United States, with at least 40 million Americans running on a regular basis.^1^ Running is a boon to wellness, with benefits to cardiovascular fitness, metabolism, adiposity, and postural balance.^2^ For many patients, running is part of their strategy to remain healthy, and each additional 15 minutes of daily exercise confers a 4% all-cause mortality reduction.^3^ It is important to both recognize and understand running and the unique activity demands and injury risks associated with runners.^4^

In the general population, there is an incidence of meniscal tear requiring meniscectomy of 61 per 100,000 individuals per year.^5^ A magnetic resonance imaging study of asymptomatic marathon runners found no increased prevalence in meniscal injury relative to controls.^6^ There is no proven correlation between running and meniscal injury, or between running and degenerative knee disease, although total running distance (greater than 40 miles per week) and history of previous injury are thought to confer a greater risk of running injury.^4^ Nonetheless, many patients sustain acute meniscal injuries during their activities whereas other patients present with sequelae of degenerative meniscal tears.

Arthroscopic partial meniscectomy (APM) is among the most commonly performed arthroscopic procedures,^7^ growing in frequency by 49% over a 10-year period.^7^ APM may appeal to runners with meniscal injuries who have exhausted nonoperative management because it is a relatively low-morbidity, outpatient procedure with a short rehabilitation period until release to full activity. However, little is known about whether or not, and at which level, runners are able to return to their former level of running if they choose to undergo APM. This study sought to determine whether, and at which frequency, runners return to running after undergoing APM. The a priori hypothesis was that 50% of patients would return to running at or more frequently than their previous frequency after undergoing APM.

## Methods

### Study Design

Institutional review board approval (2011P002663) and informed consent from patients were obtained for this study. Data from patients who underwent APM were prospectively collected in the Surgical Outcome System (Arthrex, Naples, FL) registry, which is a Health Information Portability and Accountability Act–compliant global database. The inclusion criteria were all patients who underwent a primary APM of the medial and/or lateral meniscus with or without chondroplasty by a single surgeon (E.G.M.) at a single academic medical center and met this study’s definition of a runner. Exclusion criteria were nonrunners (defined as running less than 2 times per week), incomplete preoperative or 1-year postoperative follow-up data, repeat knee arthroscopy, meniscal repair, and/or concomitant ligamentous injury.

Patients were indicated for knee arthroscopy if they had a symptomatic meniscal tear with or without chondral pathology per the clinical history, physical examination, and magnetic resonance imaging findings. Before consent for surgery, all patients had exhausted a minimum of 6 weeks of nonoperative treatments including activity modification, nonsteroidal anti-inflammatory medications, physical therapy, and/or steroid injections.

Patients were defined as runners or nonrunners according to their response to the preoperative Marx Activity Rating Scale (MARS) question that asks how often they perform a specific activity in their healthiest and most active state in the past year, specifically the item: “Running: running while playing a sport or jogging.” The options given are “Less than one time in a month,” “one time in a month,” “one time in a week,” “two to three times in a week,” and “four or more times in a week.” Those who reported running 2 to 3 times per week or 4 or more times per week were defined as runners and subsequently included in the analysis. Exclusion criteria were nonrunners (defined as running less than 2 times per week), incomplete preoperative and/or 1-year postoperative follow-up data, repeat knee arthroscopy, meniscal repair, and/or concomitant ligamentous injury.

Demographic and clinical characteristics were reviewed for the study population. Validated clinical outcome instruments were used including the MARS,^8^ Knee Injury and Osteoarthritis Outcome Score (KOOS)^9^ including its Pain and Function in Sport/Recreation components, Veterans Rand 12-Item Health Survey (VR-12)^10^ including its physical and mental components, and visual analog pain scale (VAS).^11^^,^^12^ The surgeon-recorded intraoperative findings included presence of meniscal tear (compartment involved) and severity of cartilage damage (according to Outerbridge grade^13^).

### Statistical Analysis

The primary end points of this study were (1) return to running at least twice weekly or (2) return to running at the same frequency or more frequently than preoperative baseline following APM. Means, standard deviations, and medians are presented for continuous variables. Number and percentage are presented for categorical variables. Overall change in patient-reported outcomes from preoperative baseline to 1- and 2-year postoperative follow-up intervals was computed and assessed with the paired *t* test. Means, between-group differences, and 95% confidence intervals were computed to compare change in patient reported outcomes over 1 and 2 years between those participants who did and did not return to running at 1year. The association between baseline characteristics and return to running was assessed using the unpaired *t* test or Wilcoxon rank sum test for continuous predictors and a χ^2^ test for categorical predictors, using the 1-year postoperative follow-up data. We used multivariable Poisson regression with robust error variance to assess the adjusted association between return to running and baseline characteristics, including those variables that were significantly associated with return to running in bivariate analysis. All *P* values less than .05 were considered statistically significant. All statistical analyses were performed using SAS, version 9.4 (SAS Institute, Cary, NC).

## Results

### Demographic and Clinical Characteristics

A total of 704 patients were identified for inclusion. Thirty-nine were missing baseline Marx Activity question 1, 70 were missing Marx Activity question 1 at 1-year postoperatively, and 410 patients reported running fewer than 2 times per week and were excluded, leaving 185 patients for inclusion in the study (Table 1). The study population had a mean age of 46.3 ± 11.9 years, a mean body mass index (BMI) of 28.8 ± 6.2, and was 48% female. Of these, 35% and 37% qualified as overweight or obese, respectively. Symptoms were present on average for 5.7 ± 6.8 months before the time of surgery. Seventy-nine percent underwent APM of the medial compartment and 21% of the lateral compartment. Sixty-eight percent underwent chondroplasty to address concomitant focal cartilage lesions, with a high-grade (Outerbridge grade III or IV) focal chondral lesion in at least 1 compartment in 44% of patients.

**Table 1:** Demographic and Clinical Characteristics of the Study Population

Characteristics	Mean (SD) or n (%)
Age, y	46.3 (11.9)
Sex	
Female	89 (48%)
Male	95 (52%)
Missing	1
Ethnicity (Hispanic or Latino)	
No	179 (97%)
Yes	6 (3%)
Race	
Black or African American	8 (4%)
White	168 (92%)
Other	6 (3%)
Missing	3
BMI	28.8 (6.2)
BMI group	
Normal weight (<25)	51 (28%)
Overweight (25-30)	63 (35%)
Obese (≥30)	66 (37%)
Missing	5
Duration of symptoms, mo	5.7 (6.8)
Partial meniscectomy	
Lateral	39 (21%)
Medial	146 (79%)
Chondroplasty	
No	60 (32%)
Yes	125 (68%)
Outerbridge grade	
Grade 0	51 (28%)
Grade I	15 (8%)
Grade II	38 (21%)
Grade III	39 (21%)
Grade IV	42 (23%)

Level	N (%) at 1 year after surgery	N (%) at 2 years after surgery
Preoperative baseline frequency of running		
2 or 3 times in a week	113 (61%)	98 (64%)
4 or more times in a week	72 (39%)	54 (36%)
Postoperative frequency of running		
Less than one time in a month	57 (31%)	47 (31%)
One time in a month	11 (6%)	12 (8%)
One time in a week	25 (14%)	22 (14%)
2 or 3 times in a week	62 (34%)	49 (32%)
4 or more times in a week	30 (16%)	22 (14%)
Return to running at least twice weekly		
No	93 (50%)	81 (53%)
Yes	92 (50%)	71 (47%)
Return to running at same frequency or more frequently		
No	110 (59%)	95 (63%)
Yes	75 (41%)	57 (38%)

NOTE. Patient-reported frequency of running, using the Marx Activity Rating Scale (MARS), at preoperative baseline, 1, and 2 years after APM.

APM, arthroscopic partial meniscectomy; BMI, body mass index; SD, standard deviation.

### Return to Running

Sixty-one percent of the runners included in this study reported running 2 to 3 times weekly while 39% ran 4 or more times weekly at preoperative baseline (Table 1). One year after surgery, patients returned to running 4 or more times weekly (16%), 2 to 3 times weekly (34%), once weekly (14%), once monthly (6%), or less than once monthly (31%). At 1 year, 50% of runners returned to running at least twice weekly, whereas 41% returned to running at the same frequency or more frequently than before surgery. Two years after surgery, patients returned to running 4 or more times weekly (14%), 2 to 3 times weekly (32%), once weekly (14%), once monthly (8%), or less than once monthly (32%). At 2 years, 47% of runners returned to running at least twice weekly, whereas 38% returned to the same frequency of running or more frequently than before.

### Patient-Reported Outcomes

Overall, the analytic cohort experienced significant improvements in VAS, KOOS (Pain and Function in Sport and Recreation), and the VR-12 physical component over 1 and 2 years (Table 2). The cohort did not demonstrate significant changes in the VR-12 mental component.

**Table 2:** Clinical Outcome Scores at Preoperative Baseline and 1- and 2-Year Postoperative Follow-Up

Label	Mean (SD)	Mean Δ from BL	Mean Δ from BL (95% CI)	*P* Value
Preoperative VAS	4.5 (2.1)			
Postoperative VAS (1 year)	1.5 (2.0)	–3.0	(–3.4, –2.7)	<.0001
Postoperative VAS (2 years)	1.3 (1.8)	–3.2	(–3.6, –2.8)	<.0001
Preoperative KOOS (Pain)	54.7 (14.8)			
Postoperative KOOS (Pain) (1 year)	83.0 (18.5)	28.5	(25.8-31.1)	<.0001
Postoperative KOOS (Pain) (2 years)	85.7 (15.9)	30.6	(27.5-33.7)	<.0001
Preoperative KOOS (Sport/Recreation)	29.1 (22.3)			
Postoperative KOOS (Sport/Recreation) (1 year)	70.4 (29.0)	41.6	(37.3-45.9)	<.0001
Postoperative KOOS (Sport/Recreation) (2 years)	75.4 (25.9)	45.1	(40.1-50.2)	<.0001
Preoperative VR12-P	34.1 (8.9)			
Postoperative VR12-P (1 year)	49.0 (9.2)	15.0	(13.5-16.6)	<.0001
Postoperative VR12-P (2 years)	50.2 (8.1)	16.3	(14.6-18.0)	<.0001
Preoperative VR12-M Component	55.8 (9.4)			
Postoperative VR12-M (1 year)	55.1 (8.9)	–0.6	(–2.1, 0.8)	.3964
Postoperative VR12-M (2 years)	55.5 (7.3)	–1.0	(–2.6, 0.6)	.2278

BL, baseline; CI, confidence interval; Δ from BL = change from baseline; KOOS, Knee Injury and Osteoarthritis Outcome Score; VAS, visual analog pain scale; VR12-M, Veterans RAND 12-item mental component; VR12-P, Veterans RAND 12-item physical component.

### Demographic and Clinical Predictors of Return to Running

BMI was the only demographic characteristic that predicted return to running 2 or more times weekly (*P* = .0141) (Table 3). Specifically, patients with a BMI less than 25 were significantly more likely to return than those who were obese (65% vs 39%). In addition, as compared with their counterparts who reported running 2 to 3 times weekly before surgery, patients who reported running 4 or more times weekly before surgery were significantly more likely to return to running 2 or more times weekly (60% vs 43%; *P* = .0300). Age (*P* = .1624), sex (*P* = .9962), medial versus lateral partial meniscectomy (*P* = .5628), and Outerbridge grade (*P* = .2040) were not significant predictors of returning to running 2 or more times weekly. Lower BMI was associated with return to running at the same level or more frequently than before surgery (*P* = .0327) (Table 3). Age, sex, and baseline running frequency were not associated with this return-to-running definition. In multivariable models assessing returning to running 2 or more times weekly, both BMI group (*P* = .0335) and preoperative running frequency (*P* = .0421) remained significantly associated with outcome.

**Table 3:** Association Between Baseline Characteristics and Return to Running, as Defined by Running at Least Twice Weekly and as Defined by Running at the Same Frequency or More Frequently Than Preoperative Baseline

Label	Year 1 – Return to Running ≥2 Times Per Week		*P* Value	Year 1 – Return to Running at Same Frequency or More Frequently		*P* Value
	No∗	Yes∗		No∗	Yes∗	
Age, y	47.5 (12.1)	45.1 (11.6)	.1624	46.6 (12.1)	46.0 (11.6)	.7311
Sex			.9962			.9505
Female	45 (51%)	44 (49%)		53 (60%)	36 (40%)	
Male	48 (51%)	47 (49%)		57 (60%)	38 (40%)	
BMI	29.9 (7.1)	27.7 (5.1)	.0141	29.6 (6.7)	27.7 (5.3)	.0327
BMI group			.0248			.0368
Normal weight (<25)	18 (35%)	33 (65%)		23 (45%)	28 (55%)	
Overweight (25-30)	32 (51%)	31 (49%)		39 (62%)	24 (38%)	
Obese (>30)	40 (61%)	26 (39%)		45 (68%)	21 (32%)	
Meniscectomy			.5628			.6624
Lateral	18 (46%)	21 (54%)		22 (56%)	17 (44%)	
Medial	75 (51%)	71 (49%)		88 (60%)	58 (40%)	
Outerbridge grade			.2040			.6015
Grade 0	27 (53%)	24 (47%)		29 (57%)	22 (43%)	
Grade I	4 (27%)	11 (73%)		7 (47%)	8 (53%)	
Grade II	17 (45%)	21 (55%)		22 (58%)	16 (42%)	
Grade III	24 (62%)	15 (38%)		27 (69%)	12 (31%)	
Grade IV	21 (50%)	21 (50%)		25 (60%)	17 (40%)	
Preoperative VAS	4.8 (2.2)	4.3 (2.1)	.0942	4.7 (2.2)	4.2 (2.1)	.1442
Preoperative VR12-P	32.4 (9.6)	35.7 (7.7)	.0104	32.9 (9.5)	35.8 (7.7)	.0328
Preoperative VR12-M	54.4 (10.4)	57.2 (8.0)	.0916	54.7 (10.4)	57.3 (7.4)	.1830
Pre-treatment MARS running category			.0300			.3273
2 or 3 times weekly	64 (57%)	49 (43%)		64 (57%)	49 (43%)	
4 or more times weekly	29 (40%)	43 (60%)		46 (64%)	26 (36%)	

BMI, body mass index; MARS, Marx Activity Rating Scale; VAS, visual analog pain scale; VR12-M, Veterans RAND 12-item mental component; VR12-P, Veterans RAND 12-item physical component.

∗ Mean (SD) or n (%).

### Association Between Clinical Outcome Instruments and Return to Running

Preoperative VR-12 physical component was significantly associated with return to running 2 or more times weekly (*P* = .0104). Participants who returned to running 2 or more times weekly reported more improvement in VAS score from preoperative baseline to 1 year postoperatively (*P* = .0358), KOOS (Pain) subscores from preoperative baseline to one year postoperatively (*P* = .0108), KOOS (Function in Sport and Recreation) subscores from preoperative baseline to one year postoperatively (*P* = .0096), and VR-12 physical component from preoperative baseline to one year postoperatively (*P* = .0078) also were associated with return to running (Table 4). Preoperative VR-12 physical component was significantly associated with return to running at preoperative frequency or greater (*P* = .0104), whereas changes in clinical outcome scores were not significantly associated with this return-to-running definition (Table 4).

**Table 4:** Association Between Change in Clinical Outcome Scores From Baseline and Return to Running, as Defined By Running at Least Twice Weekly and as Defined by Running at the Same Frequency or More Frequently Than Preoperative Baseline

Label	Year 1 – Return to Running ≥2 Times Per Week		*P* Value	Year 1 – Return to Running at Same Frequency or More Frequently		*P* Value
	No∗	Yes∗		No∗	Yes∗	
Baseline to Y1 Change in VAS	–2.6 (2.9)	–3.4 (2.0)	.0358	–2.8 (2.8)	–3.4 (2.0)	.0644
Baseline to Y1 Change in KOOS (Pain)	25.0 (20.7)	31.9 (15.0)	.0108	26.9 (20.4)	30.7 (14.6)	.1385
Baseline to Y1 Change in KOOS (Sport/Recreation)	35.6 (31.8)	47.0 (22.9)	.0096	38.9 (30.9)	45.4 (23.2)	.1247
Baseline to Y1 Change in VR12-P	13.0 (12.0)	17.1 (8.3)	.0078	13.9 (11.6)	16.6 (8.5)	.0701
Baseline to Y1 Change in VR12-M	–1.2 (11.1)	–0.1 (8.9)	.4865	–1.0 (11.0)	–0.0 (8.6)	.4892
Baseline to Y2 Change in VAS	–2.9 (2.6)	–3.5 (2.1)	.1942	–3.1 (2.6)	–3.4 (2.1)	.3758
Baseline to Y2 Change in KOOS (Pain)	30.6 (19.8)	30.6 (16.5)	.9896	30.8 (19.1)	30.3 (16.9)	.8860
Baseline to Y2 Change in KOOS (Sport/Recreation)	43.5 (32.0)	46.5 (24.4)	.5649	44.4 (31.8)	46.1 (22.9)	.7368
Baseline to Y2 Change in VR12-P	15.6 (11.9)	17.0 (7.9)	.4346	16.1 (11.5)	16.5 (7.7)	.8238
Baseline to Y2 Change in VR12-M	–0.7 (11.5)	–1.3 (7.5)	.7473	–1.0 (11.1)	–1.1 (7.2)	.9545

KOOS, Knee Injury and Osteoarthritis Outcome Score; SD, standard deviation; VAS, visual analog pain scale; VR12-M, Veterans RAND 12-item mental component; VR12-P, Veterans RAND 12-item physical component, Y1, year 1; Y2, year 2.

∗ Mean (SD).

## Discussion

In this study, we found that 50% of runners returned to running at least twice weekly, whereas 41% of runners returned to running at the same frequency or more frequently than their preoperative baseline. This information may be useful when counseling and indicating runners with symptomatic meniscal tears who wish to continue their active lifestyle.

Runners exhibited global improvements from preoperative baseline in validated clinical outcome instruments including KOOS (Pain) subscores, KOOS (Sport/Recreation) subscores, VAS score, and VR-12 physical component. BMI (*P* = .0248) and greater baseline running frequency (*P* = .0300) were significantly associated with return to running at year 1. Interestingly, neither pain (according to VAS score or KOOS subscores) nor burden of degenerative chondral wear (according to Outerbridge grade) accounted for inability to return to the prior frequency of running. There was likewise no significant difference for medial versus lateral partial meniscectomy.

Our study adds to the body of literature on athletes who undergo surgical treatment of meniscal tears. Nawabi et al.^14^ showed that elite professional soccer players were significantly more likely to return to their preinjury level of competition, and within a shorter interval, if they underwent medial meniscectomy than if they underwent lateral meniscectomy. In contrast, Kim et al.^15^ found that athletes returned to sport significantly faster after lateral versus medial meniscectomy, with more patients in the latter group exhibiting postoperative pain and/or effusion. Age, activity level, and extent of resection further predicted time to return. Aune et al.^16^ found that 61% of National Football League players undergoing partial lateral meniscectomy were able to return to their previous level of competition, with a significant correlation to position (nonspeed position vs speed-position) and preoperative level of competition (starter vs nonstarter).^16^

One strength of this study is the use of multiple validated clinical outcome instruments, including the MARS, which ensured inclusion of runners in our study according to standardized definitions. Furthermore, our interpretation of postoperative outcome scores in the context of a preoperative baseline, which may significantly vary between individuals, achieves normalization and minimizes selection bias.^17^ The Hawthorne effect, which dictates that study participant behavior may be altered due to the awareness of being observed, was minimized by electronically collecting bulk data en masse for this study without specific identification of the study hypothesis. Future studies may improve our understanding of why at least half of runners undergoing APM do not return to their baseline frequency of running. Possibilities include meniscal tear characteristics, extent of meniscal resection, degenerative wear present within the compartment, or other unrecognized factors. Additional research may elucidate the role of tailored postoperative rehabilitation programs and graduated return-to-running protocols in correcting specific kinematic and/or functional deficiencies in patients who struggle to return to their previous frequency of running. Medial and lateral partial meniscectomies are associated with similar rates of return to running. Compared with their preoperative baseline, runners experience significant symptomatic and functional improvement after undergoing APM for symptomatic, activity-limiting meniscal injury.

### Limitations

There are noteworthy limitations to this study. We did not independently collect data on time to return to running, running intensity and level, or running duration, all of which might further contextualize our findings. It is possible that some runners voluntarily self-regulate their postoperative frequency of running or incorporate nonimpact cross-training into their regimen to protect their remaining meniscus. Furthermore, we did not study the influence of meniscal tear size, pattern, or chronicity, or extent of meniscal resection. For instance, degenerative, complex meniscal tears are associated with chondral disease over 85% of the time,^18^ and might ostensibly have a poorer prognosis than acute tear patterns for return to running. In addition, extent of meniscal resection is known to be proportionally correlated with increased tibiofemoral contact pressures of up to 80% to 90%, and directly affects knee biomechanics and kinematics.^19^, ^20^, ^21^, ^22^, ^23^ Extent of meniscal resection may also modulate functional outcomes by altering knee kinematics, including increased knee adduction, flexion, and extension moments,^24^, ^25^, ^26^ as well as knee extensor strength.^27^, ^28^, ^29^

## Conclusions

Roughly 1 in 2 runners return to their preoperative running frequency after undergoing APM. Obesity and lower baseline running frequency are significant risk factors for inability to return to running.

## Supplementary Data

ICMJE author disclosure forms
